# Global Perspective on Kidney Transplantation: Bosnia and Herzegovina

**DOI:** 10.34067/KID.0000000000000445

**Published:** 2024-04-15

**Authors:** Enisa Mesic, Alexander Woywodt, Mirna Aleckovic-Halilovic

**Affiliations:** 1 Department of Nephrology, Dialysis and Transplantation, University Hospital Tuzla, Tuzla, Bosnia and Herzegovina; 2 Department of Renal Medicine, Lancashire Teaching Hospitals NHS Foundation Trust, Preston, United Kingdom

**Keywords:** kidney transplantation, organ transplant, renal transplantation, transplantation

## Introduction

Bosnia and Herzegovina (BiH) is a country in the West Balkans with a population of 3.5 million. BiH gained independence after the collapse of the Second Yugoslavia and the 4-year war that ended with the Dayton Agreement in 1995. The agreement implied the formation of an unusual state structure composed of distinct substate “entities,” namely the *Federation* of BiH (FBiH, consisting of ten cantons with a predominantly Muslim–Catholic population), the Republika Srpska (RS, centralized, predominantly orthodox population), and the Brčko District (a mixed population). Many important issues, including health care, are devolved to a regional level with little input from the central government. BiH is a country with medium high income and a gross domestic product of 7585.4$/per inhabitant in 2022. Dialysis is funded by state insurance and hemodialysis dominates, although peritoneal dialysis programs also exist.^1^ According to European Renal Association (ERA) registry data,^2^ 428 patients started dialysis in 2021 (121 pmp), with diabetic nephropathy as the most common underlying diagnosis, followed by hypertension and GN. The number of prevalent patients on dialysis in the same year was 2392 (680 pmp), which is comparable with similar Eastern European countries, such as Serbia (869 pmp) and Croatia (647 pmp). Many patients present late in the course of the disease, resulting in a small number of renal biopsies compared with elsewhere. Until relatively recently, Balkan nephropathy^3^ was responsible for a significant share of patients, but the incidence of this condition has declined markedly, and in 2021, only 23 of 428 patients on incident dialysis carried this diagnosis. All 29 dialysis centers send data to a national registry that contributes to the European Renal Registry, and the rate of incident patients (unadjusted) is currently around 121 pmp. The 1992–1995 war^4^ and post-Dayton governance structures continue to have a major effect on health care,^5^ and renal care and transplantation are no exception. Around 200 transplants had been performed during 1974–1991, but all activities came to a halt with the outbreak of war in 1992 until transplantation resumed in the late 1990s. Three hundred sixty-four transplants have been performed during 1997–2023 (309 from live donors, 55 deceased). We provide a primer on kidney transplantation in our divided country. We describe the legal framework and funding, provide an overview of the transplant activity from live and deceased donors, and describe some of the main challenges in this area and possible strategies to overcome them.

## Legal Framework, Governance, and Funding

During the time of the Socialist Federal Republic of Yugoslavia (1945–1992), kidney transplantation was initially only available to a small number of patients and a national waiting list did not exist. Jugotransplant was founded in 1973 as a nationwide organization to facilitate organ donation, but the overall number of transplants remained small.^6^ From the present-day viewpoint, organ donation law during the later years of the Federal Republic of Yugoslavia was remarkably progressive because it included the concept of presumed consent as early as the 1980s. This legislation applied nationwide. It was certainly unusual at the time with only Austria and Belgium choosing a similar approach in 1982 and 1986, respectively.^7^

The legal framework in present-day BiH is complicated by the unusual governance arrangements as a result of the 1994 Washington Agreement and the subsequent Dayton Peace Treaty in November 1995. Through this treaty, BiH was set up as a country with two substate autonomous entities, *i.e*., FBiH, RS, and a third unit, the Brčko District. Each of these substate units has its own ministry of health, and there is minimal national oversight. Each substate entity has its own laws and regulations on organ and tissue transplantation, which are all based on presumed consent for donation but include a clause on mandatory consent from family members. The lack of national oversight and coordination for certainly hampers cooperation between the three transplant centers Banja Luka (RS), Sarajevo (FBiH), and Tuzla (FBiH) and impairs progress in organ donation and transplantation overall. Even within the substate, it is difficult to work together in transplant medicine. Even within the substate unit of FBiH, it is difficult to reach an agreement among the ten cantons (districts), and even more so with the other substate entities. The absence of national oversight also leads to a lack of nationally agreed-upon quality criteria, and there is no agreed certification process for transplant centers, nor any national audit of outcomes. At present, the outcome of transplantation in the country is thought to be acceptable with graft survival rates of 88% at 1 year and 81% at 5 years. For comparison, according to 2021 ERA registry data, the 5-year adjusted graft survival rate for the cohort 2012–2016 was 81.4%.^2^ Detailed data about annual transplant activity are also lacking and would probably require a national agency or registry as well. Without such national governance, it is difficult to imagine a level of transplant activity that would be sufficient for membership in Eurotransplant at least in the near future.

All transplants performed in transplant centers in BiH, as well as all costs of donor processing and maintenance, as well as post-transplant care, are free and financed by state insurance. This is funded through mandatory deductions from salary plus employer contributions. Total health expenditure to gross domestic product is 9.1% in BiH, which is high for a mid-income country.

However, transplants performed abroad (most often Belarus, Russia, Turkey, Italy, France, Serbia, and Croatia) are a gray area when it comes to financing. In essence, such patients are forced to collect money from friends and family or crowdfund transplantation abroad, although some of the patients manage to get part of the funding from state insurance. These are typically patients who have a donor of a different blood group, an altruistic donor, highly sensitized patients, and patients from the RS entity. Patients who, in addition to BiH citizenship, also have Croatian citizenship can register on the waiting list in Croatia and be transplanted in a relatively short period. Both the FBiH and the RS have regulations that attempt to resolve transplant treatment abroad, where insurance institutions, *i.e*., ministries of health, finance the entire, or more often partial, treatment. Some of these activities have raised concern as to whether they are compatible with the Istanbul Declaration,^8^ although the purchase of organs and associated activities are prohibited by law in BiH. Historically, many patients were referred to hospitals abroad where there was an ABO blood group-incompatible donor because such a program did not exist within the country. However, more recently, patients are increasingly making their own arrangements for live donor transplantation with private transplant centers abroad. Many clinicians feel that in these live donor transplants, the relation of the donor and the recipient are not scrutinized sufficiently and there is increasing concern that some of these organs may have been donated in exchange for money. Another fear is that patients often return heavily immunosuppressed and with various problems and scarce written medical information, which adds further pressure to a limited post-transplant infrastructure in the country. Unfortunately, there are no exact data on how many live donor transplants have been performed abroad but nationwide it is thought that there may be around 150 such patients.

## Kidney Transplantation from Live Donors

Pre-1992 live donor transplantation was well established in Sarajevo with a dedicated transplantation institute that performed around 200 such transplants in the period 1974–1991.^9^ A small number of patients from the current territory of BiH were also transplanted in other centers in the Second Yugoslavia, mostly in Ljubljana (Slovenia) and Rijeka (Croatia). After the 1992–1995 war, the Sarajevo transplantation institute ceased to exist. In 1997, in Sarajevo, at the then State Hospital (former Military Hospital), nine transplants were performed from a living relative donor, but that project was soon terminated because the hospital did not receive a permit for transplant medicine from the Ministry of Health. In 1999, living related kidney transplantation began in Tuzla (University Hospital, Northeastern Bosnia) and, 2 years later, in Sarajevo (University Hospital). In 2010, kidney transplants from living unrelated donors began in Tuzla, and Sarajevo followed. There were ten preemptive transplantations performed in Tuzla since 2010 (on average 1 per year) and two in Sarajevo. From 1997 to the end of 2023, 278 transplants from living related and unrelated donors were performed in the FBiH (12 per year). Live donor transplantation also began at the University Hospital in Banja Luka (since 2010), and by 2019, 31 kidney transplants had been performed (3–4 per year). For transplants from living donors, donations from relatives and in-laws (including fourth-generation cousins) are allowed. Altruistic donation is not currently allowed, nor are paired transplants, because the law in both substate entities did not provide for these options. However, more recently there has been a renewed interest in altruistic and paired transplants, and there are early-stage initiatives to legalize this approach.

## Waiting List and Transplantation from Deceased Donors

A waiting list for transplantation currently only exists in one of the two substate entities, FBiH with currently approximately 200 patients (2023 data).The other two substate entities do not have a waiting list, which represents a considerable barrier toward progress with deceased donation. Maintenance of the waiting list in the FBiH is financed by the Ministry of Health. However, official waiting lists have never been established in the RS or in the Brčko District. Kidney transplants from deceased donors were first performed in Tuzla and Sarajevo in 2006 and 2007. As in many mid-income countries, this program has not grown very much as expected, and so far only a small number of such transplants have been performed with a total of around 55 recipients. At present, deceased donation is only from donors after brain death and transplantation from donors with only circulatory death does not exist.

The Law on Transplantation and Organ Donation regulates the diagnosis of brain death in accordance with modern practice elsewhere. A trained group of anesthesiologists and neurologists follow established protocols. Overall, the process is hampered by the lack of an established transplant coordinator role so that this role is performed mostly by anesthesiologists, together with their everyday roles and without allocated time or funding. Histocompatibility testing established in all three transplant centers with varying levels of support from abroad in terms of quality control and expertise. The lack of donation from deceased donors is despite the considerable enthusiasm and prolonged efforts of medical professionals, especially nephrologists, but also the support of international organizations and groups, especially neighboring Croatia.^10^

Not surprisingly therefore, the ERA registry has BiH at the bottom of the ranking in terms of the number of transplants (3 pmp), together with neighboring Serbia and Montenegro (3 pmp). The number of transplants from a deceased donor is minimal (1 pmp) and comparable with other countries of the Western Balkans, including Albania and Kosovo. Although it is noticeable that the trends in the number of transplants vary over time in most European countries, there is still a significant gap between Western and Eastern European countries, especially when it is taken into account that the number of patients with CKD is higher in Eastern Europe.^11^

**Figure 1:**
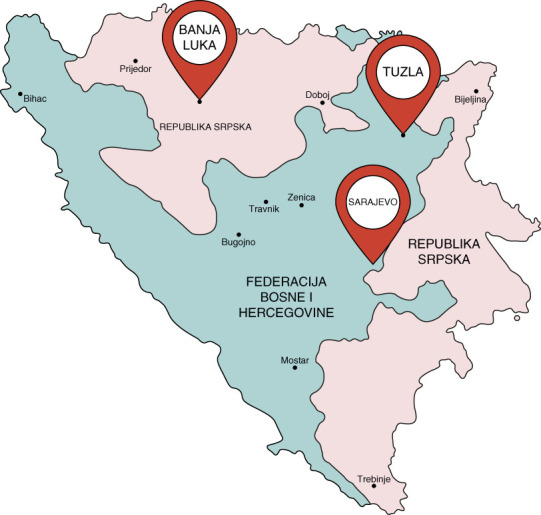
**Geographical and political map of BiH.** BiH, Bosnia and Herzegovina.

The development of kidney transplantation in BiH is intertwined with the time line of the region (Figure 1), leading to a landscape of complex challenges (Table 1). Out of all these challenges and proposed solutions, we think a national transplant authority or agency, development of a dedicated transplant coordinator role, and more cooperation with experts abroad should take priority in the near to mid-term future. All activities around organ donation are hampered by the unusual governance arrangements. Coordination and oversight at the national level and a registry should, therefore, be key priorities going forward. Staff shortages and lack of educational opportunities also play a role, and mentorships with transplant centers abroad ^13^ have been established to grow and develop local expertise. We emphasize the beneficial role of these schemes and the fact that both sides have benefited: In our experience, all experts from partnering centers abroad have found teaching in BiH a thought-provoking, interesting, and rewarding experience. Going forward, development of deceased donation with the required infrastructure and staff will not just be a question of funding but also require creativity and enthusiasm at the local level.

**Table 1:** Unique challenges to kidney transplantation in Bosnia and Herzegovina and proposed solutions

Challenge	Possible Solution
Transplantation is not coordinated at the level of the national government, and a total of 13 ministries and institutions are involved, leading to fragmented governance	Campaign for nationwide coordination of kidney transplantation under the auspices of one ministry while considering interim local and regional coordination initiatives
Lack of audit of transplantation activities and outcomes	Nationwide transplant registry
Patients having transplants abroad supported by an opaque mix of self-funding and reimbursement by state insurance	Campaign for more transparency. National transplant registry should include transplants abroad
Staff shortage in intensive care medicine and anesthesiology affecting deceased donation	Education on deceased donation and promotion of the concept starting with large hospitals; training opportunities and fellowships both in BiH and abroad. Dedicated organ donation lead roles within departments
Limited funding to develop transplant expertise within BiH	Harness goodwill of the regional and international transplant community, develop regional cooperation^12^ and links with experts abroad
Lack of dedicated transplant coordinators for deceased donation	Employ dedicated transplant coordinators and develop their role

BiH, Bosnia and Herzegovina.

## Supplementary Material
