# Supplementary material for: Global Perspective on Kidney Transplantation: Bosnia and Herzegovina
Source: Kidney360. 2024 Apr 15;5(6):903–6. doi: 10.34067/KID.0000000000000445 (PMC11219106; doi:10.34067/KID.0000000000000445)
Supplement: Supplementary file 1 [file kidney360-5-903-s001.pdf]

ASN
American Society of Nephrology
JASN
Journal of the American Society of Nephrology
KIDNEY360
Accessing Our World From Every Angle
CJASN
Clinical Journal of the American Society of Nephrology

ASN Journal Disclosure Form

As per ASN journal policy, I have disclosed any financial relationship or commitment held by myself and/or my spouse/partner in the past 36 months as included below. I have listed my Current Employer below to indicate there is a relationship requiring disclosure. If no relationship exists, my Current Employer is not listed.

M. Aleckovic-Halilovic reports the following:
Employer: University Clinical Center Tuzla

I understand that the information above will be published within the journal article, if accepted, and that failure to comply and/or to accurately and completely report the potential financial conflicts of interest could lead to the following: 1) Prior to publication, article rejection, or 2) Post-publication, sanctions ranging from, but not limited to, issuing a correction, reporting the inaccurate information to the authors' institution, banning authors from submitting work to ASN journals for varying lengths of time, and/or retraction of the published work.

Name: Mirna Aleckovic-Halilovic
Manuscript ID: K360-2024-000063R1
Manuscript Title: Global Perspective on Kidney Transplantation: Bosnia and Herzegovina
Date of Completion: April 3, 2024
Disclosure Updated Date: April 3, 2024

ASN American Society of Nephrology | JASN Journal of the American Society of Nephrology | KIDNEY360 Accessing Our World From Every Angle | CJASN Clinical Journal of the American Society of Nephrology

ASN Journal Disclosure Form

As per ASN journal policy, I have disclosed any financial relationship or commitment held by myself and/or my spouse/partner in the past 36 months as included below. I have listed my Current Employer below to indicate there is a relationship requiring disclosure. If no relationship exists, my Current Employer is not listed.

E. Mesic reports the following:
Employer: University Clinical Center Tuzla

I understand that the information above will be published within the journal article, if accepted, and that failure to comply and/or to accurately and completely report the potential financial conflicts of interest could lead to the following: 1) Prior to publication, article rejection, or 2) Post-publication, sanctions ranging from, but not limited to, issuing a correction, reporting the inaccurate information to the authors' institution, banning authors from submitting work to ASN journals for varying lengths of time, and/or retraction of the published work.

Name: Enisa Mesic
Manuscript ID: K360-2024-000063R1
Manuscript Title: Global Perspective on Kidney Transplantation: Bosnia and Herzegovina
Date of Completion: April 4, 2024
Disclosure Updated Date: April 4, 2024

ASN
American Society of Nephrology
JASN
Journal of the American Society of Nephrology
KIDNEY360
Accessing Our World From Every Angle
CJASN
Clinical Journal of the American Society of Nephrology

ASN Journal Disclosure Form

As per ASN journal policy, I have disclosed any financial relationship or commitment held by myself and/or my spouse/partner in the past 36 months as included below. I have listed my Current Employer below to indicate there is a relationship requiring disclosure. If no relationship exists, my Current Employer is not listed.

A. Woywodt reports the following:
Employer: Lancashire Teaching Hospitals NHS Foundation Trust; Consultancy: Adkins Consulting, Atheneum, Fieldscope, GKA, Medotus, Opinionsite, Rapidus; Honoraria: I have received remuneration for ad hoc consultancy work from Atheneum, Opinionsite, Adkins Research Group, Medotus, LLR Consulting.; and Other Interests or Relationships: I am on the Editorial Board of Clinical Kidney Journal and of BMC Nephrology; I received ISN expenses for a week of teaching in Tuzla/Bosnia Herzegovina in 2015 as part of an ISN mentorship scheme.

I understand that the information above will be published within the journal article, if accepted, and that failure to comply and/or to accurately and completely report the potential financial conflicts of interest could lead to the following: 1) Prior to publication, article rejection, or 2) Post-publication, sanctions ranging from, but not limited to, issuing a correction, reporting the inaccurate information to the authors' institution, banning authors from submitting work to ASN journals for varying lengths of time, and/or retraction of the published work.

Name: Alexander Woywodt
Manuscript ID: K360-2024-000063R1
Manuscript Title: Global Perspective on Kidney Transplantation: Bosnia and Herzegovina
Date of Completion: April 4, 2024
Disclosure Updated Date: April 4, 2024
